# Pharmacogenetics polygenic response score predicts outcomes in aspirin-treated stroke patients

**DOI:** 10.3389/fphar.2025.1519383

**Published:** 2025-04-01

**Authors:** Rui-Nan Ma, Dong Zhang, Zhi-Zhang Li, Ying Ding, Xiao-Guang Zhang, Jie Xue, Dan-Zhuoma Ci, Yue-Ying Bai, Liang Hu, Dai-Zhan Zhou, Yun-Hua Yue

**Affiliations:** ^1^ Department of Neurology, Yangpu Hospital, School of Medicine, Tongji University, Shanghai, China; ^2^ Biology Department, Boston College, Chestnut Hill, MA, United States

**Keywords:** aspirin, acute ischemic stroke, single nucleotide variant, pharmacogenetics, prognosis, personalized medicine

## Abstract

**Background:**

Aspirin is a cornerstone medication for acute ischemic stroke (AIS), but its efficacy varies significantly among individuals. This study aimed to develop a pharmacogenetic polygenic response score (PgxRS) to predict the incidence of adverse outcomes in aspirin-treated AIS patients.

**Methods:**

We conducted a retrospective study involving 828 AIS patients who received aspirin therapy. Fifteen candidate single nucleotide variants (SNPs) in genes related to aspirin’s mechanism of action, transport, metabolism, and platelet function were genotyped. The association between SNPs and the risk of unfavorable prognosis (defined as modified Rankin Scale score >1 at 90 days) was assessed using logistic regression analysis. Multivariable models incorporating SNPs and clinical factors were developed to predict adverse outcomes.

**Results:**

The rs1045642GG genotype in the ABCB1 gene was significantly associated with a lower risk of unfavorable prognosis, while the rs1371097T allele in the P2Y1 gene was linked to a higher risk. A prediction model incorporating these two SNPs along with clinical variables demonstrated moderate diagnostic accuracy for predicting unfavorable prognosis (AUC = 0.78, 95% CI: 0.74–0.81).

**Conclusion:**

Our findings suggest that rs1045642 and rs1371097 genotypes contribute to variability in aspirin response among AIS patients. The developed PgxRS, incorporating these SNPs and clinical factors, can potentially aid in risk stratification and guide personalized antiplatelet therapy decisions. However, further validation in larger, diverse cohorts is warranted.

## Introduction

Stroke is a leading cause of disability and mortality worldwide, ranking as the second most common cause of death and a significant contributor to global disability (Ma et al., 2021; Feigin et al., 2022). According to data from the World Stroke Organization (WSO), over 12 million new stroke cases occur annually, with more than 6 million stroke-related deaths reported each year (Feigin et al., 2022). Antiplatelet therapy plays a pivotal role in the management of ischemic stroke and the prevention of recurrent events (Bhatia et al., 2021). Among available treatments, aspirin has been shown to reduce the risk of recurrent stroke by approximately 15% in secondary prevention (Hankey, 2014). Consequently, aspirin remains a cornerstone therapy for the management of stroke and other cardiovascular diseases.

However, the efficacy of aspirin varies significantly among patients, with a substantial proportion experiencing recurrent events despite treatment—a phenomenon referred to as aspirin resistance or non-responsiveness (Kim et al., 2015). This variability presents a significant challenge in stroke management, adversely affecting long-term prognosis and mortality rates. While factors such as age, sex, comorbidities, and medication adherence have been identified as contributors to this variability (Yi et al., 2013), they do not fully account for the observed differences in aspirin response. Emerging evidence highlights the critical role of genetic factors in modulating aspirin efficacy (Silva et al., 2023). Variants in genes associated with aspirin’s mechanism of action, drug transport and metabolism, as well as platelet function, are believed to underlie individual differences in treatment response.

Numerous studies have demonstrated associations between genetic variants and the therapeutic efficacy of aspirin. For instance, rs1330344, located in the COX-1 gene, which encodes cyclooxygenase-1 (COX-1)—a key target of aspirin involved in prostaglandin synthesis and platelet aggregation regulation—has been linked to aspirin resistance. Specifically, the G allele at the rs1330344 site has been associated with high on-treatment platelet reactivity (HTPR) and an increased risk of cardiovascular events among carriers (Li et al., 2013). Additionally, studies have identified an interaction between rs3842787 (in the COX-1 gene) and rs20417 (in the COX-2 gene), which may significantly increase the risk of aspirin resistance in ischemic stroke patients. This interaction has been shown to independently predict aspirin resistance and is associated with reduced platelet aggregation activity (Yi et al., 2016).

Variants in other genes, including COX-2 (rs20417CC), P2Y1 (rs1371097), and GPIIIa (rs2317676), have also been linked to aspirin resistance in stroke patients (Yi et al., 2017a). These genetic variations are thought to influence platelet activation and aggregation, thereby modulating aspirin’s antiplatelet effects. Furthermore, rs1045642, located in the ABCB1 gene, encodes P-glycoprotein, a critical protein involved in drug efflux. Variants in rs1045642 have been associated with altered drug efficacy and adverse reactions, including impaired absorption of antiplatelet agents such as aspirin and clopidogrel in patients with coronary heart disease, potentially contributing to drug resistance (Taubert et al., 2006).

This study explores the association between 15 candidate single nucleotide polymorphisms (SNPs) and long-term outcomes in a cohort of 828 acute ischemic stroke patients undergoing aspirin therapy. The investigation focuses on SNPs within key pathways implicated in aspirin response, including those related to drug action, transport, metabolism, and platelet function. The primary objective is to develop a pharmacogenetic polygenic response score (PgxRS) to predict the risk of adverse outcomes in this patient population. By identifying genetic markers associated with treatment response, this study aims to advance the implementation of personalized antiplatelet therapy in stroke management, thereby improving clinical outcomes and reducing the burden of this debilitating disease.

## Materials and methods

### Study design and participants

**FIGURE 1:**
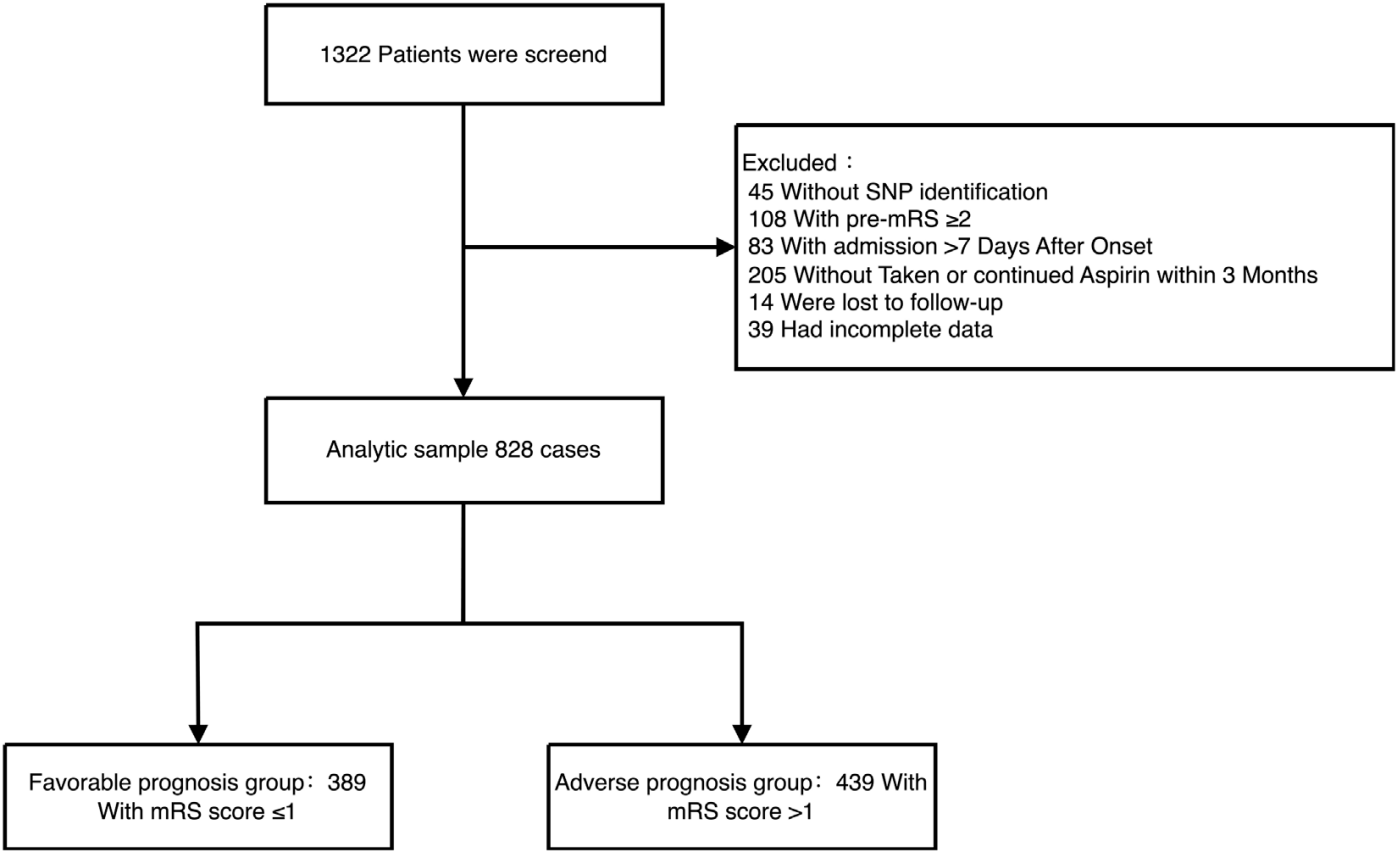
Flow chart of sample screening. This flowchart represents the data selection process for ischemic stroke patients admitted to the Department of Neurology at Yangpu District Central Hospital from September 2016 to October 2020. It shows the initial number of patients, the steps taken to screen the data, and the reasons for data exclusion, leading to the final count of effective data cases.

This single-center, retrospective study included 1,322 patients with acute ischemic stroke admitted to the Neurology Department of Yangpu District Central Hospital in Shanghai between September 2016 and October 2020. Eligible patients were those who received 100 mg of aspirin daily within 24 h of admission, continued long-term aspirin monotherapy (100 mg daily) after 21 days, and had complete clinical and genetic data available. Following the application of exclusion criteria (detailed in Figure 1), a total of 828 patients were included in the final analysis. Stroke subtypes were classified based on the TOAST (Trial of Org 10,172 in Acute Stroke Treatment) criteria. The study protocol was approved by the Ethics Committee of Yangpu District Central Hospital, and informed consent was obtained from all participants or their legal representatives.

### Outcomes evaluation

The primary outcome was functional outcome at 90 days, measured using the modified Rankin Scale (mRS). A favorable outcome was defined as mRS score ≤1 and an unfavorable outcome as mRS score >1 (Chen et al., 2024).

The secondary outcome was a composite endpoint of new clinical vascular events, including ischemic stroke, hemorrhagic stroke, myocardial infarction, or vascular death. Early neurological deterioration, defined as an increase of ≥2 points in the NIHSS score from baseline within 3 days of admission, was also assessed (Gong et al., 2024). All scores and outcome evaluations were confirmed by two or more specialized neurologists.

### DNA extraction

All patients participated in a genetic sub-study. A 3 mL blood sample was extracted from the brachial vein of each patient and collected in ethylenediaminetetraacetic acid (EDTA) tubes for preservation at −20°C. Following the manufacturer’s instructions, genomic DNA was isolated from the blood samples using magnetic bead separation with the Lab-Aid 820 Nucleic Acid Extraction Midi Kit and stored at −20°C.

### SNP selection

In this study, 15 genetic variants were selected based on findings from previous research (detailed in Table 1). These variants, along with their corresponding genes, were categorized into three functional groups: those involved in aspirin’s mechanism of action (COX-1: rs1330344, COX-2: rs20417) (Li et al., 2013; Yi et al., 2016; Yi et al., 2017a), drug transport and metabolism (ABCB1: rs1045642, rs1128503, rs4148727; CYP2C9: rs1057910, rs1799853; CYP3A5: rs776746; NR1I2: rs13059232) (Taubert et al., 2006; Pilotto et al., 2007; Li et al., 2018; Pan et al., 2019; Chen et al., 2019; Zou et al., 2020), and platelet function (P2Y12: rs16863323, rs2046934, rs9859538; P2Y1: rs1371097; GPIIIa: rs2317676, rs5918) (Yi et al., 2016; Yi et al., 2017a; Li et al., 2018; Cooke et al., 2006; Li et al., 2016; Yi et al., 2017b). All genetic variants underwent Hardy-Weinberg Equilibrium (HWE) testing.

**TABLE 1:** Major SNPs Reported for aspirin.

SNP	Gene	Participants	Ethnic group	Genetic associations and outcome	References
Mechanism of action					
rs1330344	COX-1	431 patients on aspirin treatment with ischemic stroke	Chinese	The G allele at the rs1330344 locus in the COX-1 gene is associated with aspirin resistance	Li et al. (2013)
rs20417	COX-2	850 ischemic stroke patients	Chinese	The interaction between COX-2 (rs20417CC), P2Y1 (rs1371097), and GPIIIa (rs2317676) is associated with aspirin resistance	Yi et al. (2016), Yi et al. (2017a)
Genetics variants influencing pharma codynamics					
rs1045642	ABCB1	60 patients with coronary artery disease	Germany	The ABCB1 C3435T mutation leads to impaired P-glycoprotein function, which may hinder the absorption of antiplatelet drugs such as aspirin and clopidogrel, resulting in drug resistance	Taubert et al. (2006)
rs1128503	ABCB1	157 patients on dual-antiplatelet (aspirin plus clopidogrel) treatment	Chinese	The ABCB1 rs1128503 mutation may reduce the recurrence rate of ischemic stroke events in patients with intracranial arterial stenosis	Li et al. (2018)
rs4148727	ABCB1	3,010 patients with minor stroke or TIA	Chinese	Dual antiplatelet therapy with clopidogrel plus aspirin is associated with a reduced risk of stroke recurrence in patients with the ABCB1 -154TT (rs4148727) and 3435 CC (rs1045642) genotypes, compared to aspirin treatment alone	Pan et al. (2019)
rs1057910	CYP2C9	578 patients with coronary artery disease	Chinese	The rs1057910 gene polymorphism is a risk factor for high platelet reactivity	Zou et al. (2020)
rs1799853	CYP2C9	26 patients with endoscopically documented NSAID-related gastroduodenal bleeding lesions and 56 controls	Italy	The CYP2C9*2 and *3 mutations (occurring at the R144C and I359L loci, respectively) have been reported to be associated with reduced CYP2C9 enzyme activity	Pilotto et al. (2007)
rs776746	CYP3A5	578 patients with coronary artery disease	Chinese	The rs776746 gene polymorphism is a protective factor for high platelet reactivity	Zou et al. (2020)
rs13059232	NR1I2	634 patients on aspirin or clopidogrel treatment	Chinese	The NR1I2 (rs13059232) gene polymorphism has been identified as an independent risk factor for long-term clinical prognosis in the clopidogrel cohort, but similar results were not observed in the matched aspirin cohort	Chen et al. (2019)
Genetics variants of platelet function					
rs16863323	P2Y12	426 patients with acute minor ischemic stroke	Chinese	There is a significant gene-gene interaction between the rs16863323 and rs2317676 gene polymorphisms, which are independently associated with poor antiplatelet drug responsiveness and increased risk of major cardiovascular events in patients with minor stroke	Yi et al. (2017b)
rs2046934	P2Y12	268 patients with symptomatic extracranial or intracranial stenosis	Chinese	Carriers of the rs2046934 A allele in P2Y12 are significantly associated with recurrent ischemic events	Li et al. (2016)
rs9859538	P2Y12	157 patients on dual-antiplatelet (aspirin plus clopidogrel) treatment	Chinese	The rs9859538 and rs10935842 gene polymorphisms in P2Y12 are associated with an increased likelihood of recurrent events	Li et al. (2018)
rs1371097	P2Y1	850 patients on aspirin treatment with ischemic stroke	Chinese	The interaction between the rs20417CC/rs1371097TT/rs2317676GG genotypes is associated with aspirin resistance	Yi et al. (2016), Yi et al. (2017a)
rs2317676	GPIIIa	850 ischemic stroke patients	Chinese	The platelet glycoprotein gene GPIIIa (rs2317676GG) is associated with aspirin resistance and vascular event recurrence	Yi et al. (2016), Yi et al. (2017b)
rs5918	GPIIIa	30 PlA1/A1 and 30 PlA1/A2 patients coronary artery disease	America	Patients carrying the risk allele PIA2 exhibit high platelet reactivity during aspirin treatment	Cooke et al. (2006)

### SNP genotyping

All 15 SNPs were genotyped by in-house developed multiplex tagged-amplicon deep sequencing method in our previous studies. A total 15 target-specific primer pairs were designed by using Primer3, and synthesized with common adapter sequences at their 5′ends as previously described (Zou et al., 2018). After Pre-amplification of the target amplicons, SAP-Exo1 Enzymatic PCR Cleanup, second barcoded PCR reaction, quantification and Clean-up of the DNA library, the sequencing were performed on the NovaSeq 6,000 Sequencing System. The Burrows-Wheeler Aligner (version 0.7.17) was used to map the raw paired 150 bp-long reads to the human reference genome. Local realignment, recalibrate base quality scores, calling variants and variant filtration were performed by using the Genome Analysis Toolkit (version 4.3) as previously described.

### Statistical analyses

Statistical analyses were conducted using SPSS version 22.0. The normality of continuous variables was assessed using the Kolmogorov-Smirnov test. Normally distributed data are presented as mean ± standard deviation (SD) and compared using independent t-tests, while non-normally distributed data are presented as median (interquartile range, IQR) and compared using Mann-Whitney U tests. Categorical variables are expressed as numbers and percentages (%) and analyzed using Pearson’s chi-square test or Fisher’s exact test, as appropriate.

Associations between single nucleotide polymorphism (SNP) genotypes (analyzed using additive, dominant, and recessive models) and the risk of unfavorable prognosis were evaluated using multivariate logistic regression analysis. The recessive model assumes that only homozygous mutations are associated with the trait (BB vs AA + AB), the dominant model assumes that both heterozygous and homozygous mutations are associated with the trait (AB + BB vs AA), and the additive model assumes a linear additive effect of genotype on the trait (BB vs. AB vs. AA). Confounding factors identified as significantly different between groups in univariate analysis were included in the multivariate models. Highly correlated variables were identified through correlation matrix analysis and excluded to avoid multicollinearity.

Subgroup analyses were performed using multivariate logistic regression models stratified by sex to evaluate the relationship between SNP loci and unfavorable prognosis in male and female patients separately. The Pharmacogenetics Polygenic Response Score (PgxRS) was constructed by using the β (beta) coefficients obtained from multivariate logistic regression analysis as weighting factors for the included variables. The diagnostic performance of the predictive model, which integrated SNPs and clinical factors, was assessed using receiver operating characteristic (ROC) curve analysis. The area under the curve (AUC) was calculated to evaluate the model’s discriminative ability. A p-value <0.05 was considered statistically significant for all analyses.

## Results

### Patient characteristics

Of the 828 patients included in the study, 389 (47.0%) had a favorable prognosis at 90 days (mRS score ≤1), while 439 (53.0%) had an unfavorable prognosis (mRS score >1). As shown in Table 2, patients with unfavorable prognosis were significantly older, had higher NIHSS scores on admission, and were more likely to have a history of diabetes and stroke. They were also less likely to be male and had a lower prevalence of smoking history.

**TABLE 2:** Characteristics of baseline clinical data according to prognosis group.

	Favorable prognosis group	Adverse prognosis group	P
Number of cases, n	**389**	**439**	
Age (years)	65.62 **±** 10.95	73.41 **±** 12.01	**0.00**
Age Groups			**0.00**
0–60	120 (30.85)	69 (15.72)	
61–70	155 (39.85)	122 (27.79)	
71–80	69 (17.74)	97 (22.10)	
>80	45 (11.57)	151 (34.40)	
Gender (Male, n (%))	274 (70.44)	265 (60.36)	**0.00**
Body Mass Index (BMI, kg/m^2^)	24.15 **±** 3.41	24.42 **±** 3.59	0.29
Medical history, n (%)			
Hypertension	294 (75.58)	331 (75.40)	0.98
Diabetes	112 (28.79)	157 (35.76)	**0.04**
Coronary heart disease	38 (9.77)	60 (13.67)	0.10
Stroke	63 (16.20)	111 (25.28)	**0.00**
Smoking	198 (50.90)	180 (41.00)	**0.01**
Admission NIHSS score, Median (IQR)	2 (1, 4)	4 (2, 8)	**0.00**
TOAST classification, n (%)			**0.00**
Large artery atherosclerosis	168 (43.19)	230 (52.39)	
Cardioembolic	24 (6.17)	44 (10.02)	
Small artery occlusion	173 (44.47)	147 (33.49)	
Other etiology	4 (1.03)	8 (1.82)	
Unknown etiology	20 (5.14)	10 (2.28)	
Medication during hospitalization, n (%)			0.13
Aspirin	111 (28.53)	154 (35.08)	
Aspirin + Clopidogrel	267 (68.64)	274 (62.41)	
Aspirin + Other	11 (2.83)	11 (2.51)	
Statins medication	355 (91.26)	406 (92.48)	0.61
Laboratory indicators, Mean ± SD			
White Blood Cell count (WBC, ×10^9^/L)	7.48 **±** 2.47	7.83 **±** 2.77	0.06
Red Blood Cell count (RBC, ×10^12^/L)	4.53 **±** 0.57	4.36 **±** 0.64	**0.00**
Platelet count (PLT, ×10^9^/L)	220.31 **±** 65.01	218.07 **±** 69.84	0.64
C-Reactive Protein (CRP, mg/L)	9.82 **±** 18.61	16.59 **±** 30.13	**0.00**
Neutrophil Count (NEUT#, ×10^9^/L)	5.03 **±** 2.35	5.60 **±** 2.59	**0.00**
Lymphocyte Count (LYMPH#, ×10^9^/L)	1.75 **±** 0.70	1.56 **±** 0.94	**0.00**
Creatinine (Cr, mg/dL)	74.25 **±** 44.94	75.96 **±** 60.99	0.67
Uric Acid (UA, mg/dL)	327.98 **±** 101.11	319.29 **±** 106.55	0.24
Fasting Plasma Glucose (FPG, mg/dL)	7.15 **±** 3.16	7.64 **±** 3.38	**0.03**
Glycated Hemoglobin (HbA1c, %)	6.83 **±** 1.80	7.04 **±** 1.89	0.10
Triglycerides (TG, mg/dL)	1.59 **±** 0.97	1.56 **±** 1.29	0.75
Total Cholesterol (CHOL, mg/dL)	4.80 **±** 1.21	4.84 **±** 1.30	0.68
High-Density Lipoprotein cholesterol (HDL-c, mg/dL)	1.12 **±** 0.26	1.11 **±** 0.29	0.81
Low-Density Lipoprotein cholesterol (LDL-c, mg/dL)	3.12 **±** 0.89	3.14 **±** 0.91	0.76
Homocysteine (Hcy, µmol/L)	18.25 **±** 10.84	19.66 **±** 13.84	0.11
AA Inhibition Rate(%)	66.31 **±** 23.67	68.53 **±** 24.52	0.31
Endpoint events, n (%)			
Early neurological deterioration	10 (2.57)	70 (15.95)	**0.00**
cardiovascular and cerebrovascular composite events	20 (5.14)	41 (9.34)	**0.03**
Hospital readmission for treatment	64 (16.45)	139 (31.66)	**0.00**

Note: Bold values indicate statistical significance (P < 0.05).

Laboratory findings showed that the unfavorable prognosis group had significantly higher levels of fasting blood glucose, C-reactive protein (CRP), and neutrophil count compared to the favorable prognosis group. Red blood cell count and lymphocyte count were lower in the unfavorable prognosis group (Table 2).

Regarding secondary outcomes, the unfavorable prognosis group experienced significantly higher rates of early neurological deterioration, cardiovascular and cerebrovascular events, and readmission for treatment (P < 0.05 for all comparisons).

### Genotype distribution and associations with clinical outcomes

Among the 15 SNPs analyzed (after excluding two SNPs with low mutation frequencies), the rs1045642 (ABCB1) and rs1371097 (P2Y1) genotypes showed significant differences in distribution between the favorable and unfavorable prognosis groups (Table 3).

**TABLE 3:** Characteristics of genotype data according to prognosis group.

SNPs	Ref/Alt	Favorable prognosis group (n = 389)			Adverse prognosis group (n = 439)			Allele(P)	Genotype(P)		Recessive
		AA (%)	AB (%)	BB(%)	AA (%)	AB (%)	BB(%)		Dominant	Additive	
rs1045642	A>G	98 (27.61)	126 (35.49)	131 (36.90)	105 (25.80)	185 (45.45)	117 (28.75)	0.24	0.63	**0.01**	**0.02**
rs1057910	A>C	361 (92.80)	28 (7.20)	0 (0.00)	414 (94.31)	25 (5.69)	0 (0.00)	0.47	0.46	0.46	1
rs1128503	A>G	170 (43.70)	179 (46.02)	40 (10.28)	199 (45.33)	192 (43.74)	48 (10.93)	0.87	0.69	0.8	0.85
rs13059232	T>C	64 (16.45)	181 (46.53)	144 (37.02)	74 (16.86)	211 (48.06)	154 (35.08)	0.66	0.95	0.84	0.61
rs1330344	C>T	44 (11.31)	201 (51.67)	144 (37.02)	69 (15.72)	206 (46.92)	164 (37.36)	0.42	0.08	0.14	0.98
rs1371097	C>T	209 (53.73)	154 (39.59)	26 (6.68)	206 (46.92)	188 (42.82)	45 (10.25)	**0.02**	0.06	0.06	0.09
rs16863323	C>T	96 (24.81)	231 (59.69)	60 (15.50)	103 (23.46)	256 (58.31)	80 (18.22)	0.44	0.71	0.57	0.34
rs1799853	C>T	388 (99.74)	1 (0.26)	0 (0.00)	439 (100.00)	0 (0.00)	0 (0.00)	0.95	0.95	0.95	1
rs20417	C>G	346 (88.95)	40 (10.28)	3 (0.77)	384 (87.47)	52 (11.85)	3 (0.68)	0.63	0.58	0.77	0.79
rs2046934	G>A	7 (1.80)	117 (30.08)	265 (68.12)	10 (2.28)	137 (31.21)	292 (66.51)	0.62	0.81	0.82	0.68
rs2317676	A>G	245 (62.98)	130 (33.42)	14 (3.60)	284 (64.69)	134 (30.52)	21 (4.78)	0.94	0.66	0.52	0.5
rs4148727	A>G	307 (78.92)	80 (20.57)	2 (0.51)	353 (80.41)	82 (18.68)	4 (0.91)	0.78	0.66	0.64	0.79
rs5918	T>C	385 (98.97)	4 (1.03)	0 (0.00)	436 (99.77)	1 (0.23)	0 (0.00)	0.3	0.3	0.3	1
rs776746	C>T	207 (53.21)	151 (38.82)	31 (7.97)	220 (50.11)	187 (42.60)	32 (7.29)	0.62	0.41	0.54	0.81
rs9859538	G>A	299 (76.86)	78 (20.05)	12 (3.08)	328 (74.89)	96 (21.92)	14 (3.20)	0.59	0.56	0.8	0.91

Notes:

A: Represents the reference allele (wild-type or common allele).

B: Represents the alternative allele (variant or minor allele).

AA: Homozygous genotype with two reference alleles (AA).

AB: Heterozygous genotype with one reference allele and one alternative allele (AB).

BB: Homozygous genotype with two alternative alleles (BB).

Allele (P): P-value for the difference between groups in genetic variants proportion, indicating the significance of allelic frequency differences between groups.

Genotype (P): P-value indicating the significance of differences in genotype frequencies between groups.

Note: Bold values indicate statistical significance (P < 0.05).

As shown in Table 4, in the recessive model of logistic regression analysis, the rs1045642GG genotype was significantly associated with a lower risk of unfavorable prognosis after adjusting for confounding factors (adjusted odds ratio [OR] = 0.66, 95% confidence interval [CI]: 0.48–0.92, P = 0.01 in Model 1; adjusted OR = 0.67, 95% CI: 0.49–0.93, P = 0.01 in Model 2). The rs1371097 T allele, in the additive model, was independently associated with a higher risk of unfavorable prognosis (adjusted OR = 1.27, 95% CI: 1.00–1.61, P = 0.05 in Model 1; adjusted OR = 1.30, 95% CI: 1.03–1.64, P = 0.03 in Model 2).

**TABLE 4:** Correlation between SNPs and unfavorable prognosis in ischemic stroke after adjusting for confounding factors.

	Genotype	Favorable prognosis group (n = 389)		Adverse prognosis group (n = 439)		Model1			Model2		
		n	%	n	%	OR	95% CI	P	OR	95% CI	P
rs1045642	A-G										
Dominant	AA	98	27.61%	105	25.80%	1.02	[0.72, 1.45]	0.91	1.01	[0.71, 1.44]	0.96
	GG + AG	257	72.39%	302	74.20%						
Additive	AA	98	27.61%	105	25.80%	0.88	[0.74, 1.04]	0.12	0.88	[0.74, 1.04]	0.13
	AG	126	35.49%	185	45.45%						
	GG	131	36.90%	117	28.75%						
Recessive	AA + AG	224	63.10%	290	71.25%	0.66	[0.48, 0.91]	**0.01**	0.67	[0.48, 0.92]	**0.01**
	GG	131	36.90%	117	28.75%						
rs1371097	C-T										
Dominant	CC	209	53.73%	206	46.92%	1.29	[0.95, 1.76]	0.10	1.33	[0.97, 1.81]	0.07
	TT + CT	180	46.27%	233	53.08%						
Additive	CC	209	53.73%	206	46.92%	1.27	[1.0, 1.62]	**0.05**	1.30	[1.02, 1.66]	**0.03**
	CT	154	39.59%	188	42.82%						
	TT	26	6.68%	45	10.25%						
Recessive	CC + CT	363	93.32%	394	89.75%	1.57	[0.9, 2.73]	0.11	1.63	[0.94, 2.84]	0.08
	TT	26	6.68%	45	10.25%						

Model 1: Adjusted for age, gender, history of diabetes, history of prior stroke, TOAST classification, and admission NIHSS score.

Model 2: In addition to the variables in Model 1, Model 2 also includes adjustments for fasting blood glucose, red blood cell count, C-reactive protein (CRP), neutrophil count, and lymphocyte count. The history of diabetes is excluded from Model 2.

Note: Bold values indicate statistical significance (P < 0.05).

### Subgroup analysis

We further conducted subgroup analyses in male patients (n = 539) and female patients (n = 289). The results showed that among male patients, 274 patients had a favorable functional outcome, accounting for 50.83% (Supplementary Table S3). In the recessive model, after adjusting for confounding factors, the rs1045642GG genotype was negatively correlated with adverse prognosis (adjusted odds ratio [OR] = 0.66, 95% confidence interval [CI]: 0.45–0.97, P = 0.04 in Model 1; adjusted OR = 0.67, 95% CI: 0.45–0.98, P = 0.04 in Model 2), while rs1371097 did not show any correlation with functional outcomes after adjustment for confounding factors (P > 0.05) (Supplementary Tables S5, S7).

Among female patients, 115 patients had a favorable functional outcome, accounting for 39.79% of the female patients (Supplementary Table S4). In the recessive model, after adjusting for confounding factors, the rs1045642GG genotype was negatively correlated with adverse prognosis (adjusted odds ratio [OR] = 0.66, 95% confidence interval [CI]: 0.48–0.90, P = 0.01 in Model 1; adjusted OR = 0.66, 95% CI: 0.48–0.91, P = 0.01 in Model 2). In the dominant model, after adjusting for confounding factors, the rs1371097TT + TC genotype was independently associated with an increased risk of adverse prognosis (adjusted odds ratio [OR] = 1.28, 95% confidence interval [CI]: 1.0–1.62, P = 0.05 in Model 1; adjusted OR = 1.30, 95% CI: 1.03–1.66, P = 0.03 in Model 2) (Supplementary Tables S6, S8).

### Construction of a molecular prediction model for adverse prognosis in stroke

Two multivariable logistic regression models (Model 1 and Model 2, Table 5) incorporating rs1045642, rs1371097, and relevant clinical factors were constructed to predict adverse prognosis.

**TABLE 5:** Results of multivariable logistic regression analysis for rs1045642 and rs1371097 SNP genotypes in predicting adverse prognosis in stroke.

	Beta	OR	P	0.025	0.975
const	−4.49	0.01	0.00	−5.59	−3.38
rs1045642	−0.41	0.66	0.01	−0.73	−0.09
rs1371097	0.24	1.27	0.05	0.00	0.48
sex	−0.03	0.97	0.88	−0.37	0.32
age	0.05	1.06	0.00	0.04	0.07
history of diabetes	0.45	1.57	0.01	0.12	0.78
history of stroke	0.41	1.51	0.04	0.03	0.79
TOAST classification	−0.03	0.97	0.67	−0.17	0.11
NIHSS score at admission	0.18	1.19	0.00	0.13	0.22

The table provides the coefficients, odds ratios (ORs), and p-values for the SNP, genotypes in the prediction models (Model 1 and Model 2). These values indicate the strength and significance of the association between the SNP, genotypes and adverse prognosis in stroke, after adjusting for the relevant covariates included in each model.

Adverse prognosis prediction model 1:

$$\hat{y}=\frac{1}{1+e^{—4.50-0.41x_{1}+0.23x_{2}-0.03x_{3}+0.05x_{4}+0.45x_{5}+0.18x_{6}-0.03x_{7}+0.41x_{8}}}$$

In the model, *x*1 represents the genotype of rs1045642 (0: AA, AG; 1: GG), *x*2 represents the genotype of rs1371097 (0: CC, 1: CT, 2: TT), *x*3 represents gender (0: female, 1: male), *x*4 represents age (years), *x*5 represents the history of diabetes (0: no, 1: yes), *x*6 represents the NIHSS score at admission, *x*7 represents the TOAST classification (1: large artery, 2: cardioembolic, 3: small artery occlusion, 4: other causes, 5: undetermined), and *x*8 represents the history of prior stroke (0: no, 1: yes).

**FIGURE 2:**
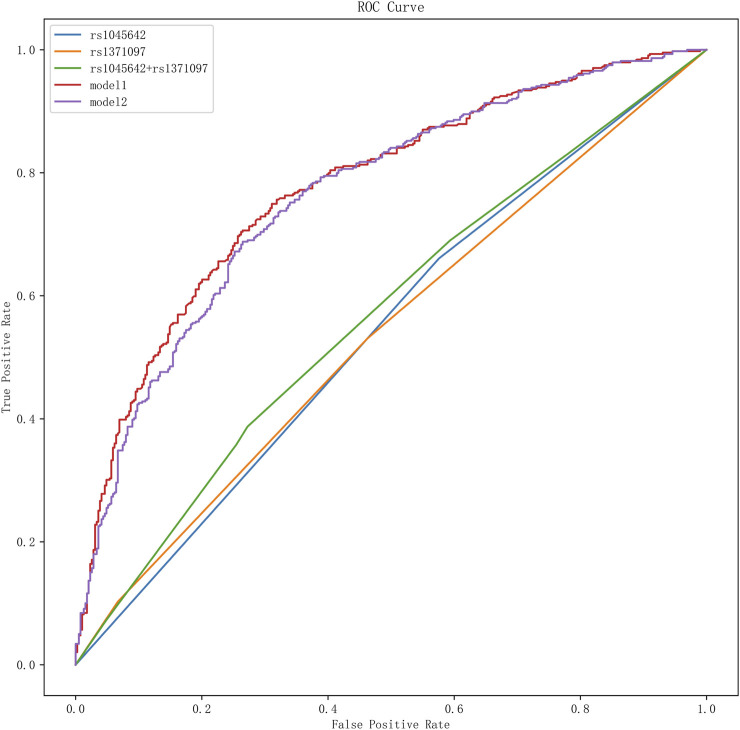
ROC curve analysis of SNPs and different models for adverse prognosis. It presents the results of the receiver operating characteristic (ROC) curve analysis for single nucleotide polymorphisms (SNPs) and various models in predicting adverse outcomes. In this graph, different curves represent the effectiveness of various SNPs and prognostic models in distinguishing adverse outcomes.

ROC curve analysis demonstrated that Model 1, with an optimal diagnostic threshold of 0.53, achieved a sensitivity of 70% and specificity of 75% in predicting unfavorable prognosis. The AUC for Model 1 was 0.78 (95% CI: 0.74–0.81, P < 0.05), indicating moderate diagnostic accuracy. Model 2, incorporating additional clinical variables, did not demonstrate superior performance compared to Model 1 (AUC = 0.78, 95% CI: 0.75–0.81, P < 0.05). The AUCs and other performance metrics are summarized in Table 6 and visually represented in Figure 2.

**TABLE 6:** Diagnostic values of the two prediction models.

	Model1	Model2
AUC	0.78	0.78
AUC Confidence Interval	[0.74,0.81]	[0.75,0.81]
Optimal Cutoff Value	0.53	0.51
Sensitivity	0.70	0.72
Specificity	0.75	0.74

## Discussion

Our study explored the impact of genetic factors on aspirin response in patients with acute ischemic stroke, focusing on 15 candidate SNPs. We identified that the rs1045642 GG genotype in the ABCB1 gene was associated with a reduced risk of unfavorable prognosis, whereas the rs1371097T allele in the P2Y1 gene was linked to an increased risk. These findings emphasize the significance of genetic variability in aspirin response and highlight potential targets for personalized antiplatelet therapy.

The ABCB1 gene encodes P-glycoprotein, a transporter protein involved in the absorption and distribution of aspirin (Thiebaut et al., 1987). Previous studies have associated the rs1045642 variant with altered P-glycoprotein function and aspirin resistance (Pan et al., 2019; Peng et al., 2016; Sharma et al., 2012). Our findings are consistent with these reports, suggesting that individuals with the GG genotype may exhibit improved aspirin absorption and potentially greater therapeutic benefit. Furthermore, it has been reported that the induction or overexpression of ABCB1 in the small intestine can mitigate aspirin-induced intestinal epithelial cell injury (Kugai et al., 2013), indicating that ABCB1 may play a role in reducing the risk of aspirin-related gastrointestinal ulcers or bleeding events. His protective effect may also contribute to improved adherence to aspirin therapy among stroke patients. These findings underscore the need for further research to elucidate the relationship between ABCB1 gene variants, aspirin resistance, and adverse gastrointestinal reactions in long-term aspirin users.

The P2Y1 gene encodes a platelet receptor that plays a critical role in platelet activation and aggregation (Storey, 2006). Previous studies have suggested that the rs1371097 variant may influence platelet reactivity to aspirin (Yi et al., 2017a; Timur et al., 2012). In a meta-analysis, Yi et al. investigated the association between COX-2, P2Y1, GPIIIa, and aspirin resistance in stroke patients, reporting that the frequency of the TT + CT genotypes of rs1371097 was significantly higher in individuals with aspirin resistance (Yi et al., 2017a). Consistent with these findings, our study demonstrated that the TT + CT genotypes of rs1371097 were independently associated with unfavorable prognosis in stroke patients. These results suggest that the rs1371097TT + CT genotypes may contribute to aspirin resistance by affecting platelet aggregation, thereby influencing stroke outcomes.

Overall, our findings are consistent with previous studies, suggesting that genetic variants in rs1045642 and rs1371097 may play significant roles in stroke prognosis. However, gender subgroup analysis revealed notable differences in the effects of these SNPs between male and female patients. Specifically, the GG genotype of rs1045642 was associated with a lower risk of adverse prognosis in both males and females, consistent with the overall analysis. In contrast, the impact of rs1371097 exhibited distinct patterns between genders: in the female subgroup, the TT + TC genotype was significantly associated with adverse prognosis, whereas no such association was observed in male patients. This gender difference persisted even after adjusting for potential confounders, further suggesting that rs1371097 may influence stroke prognosis through distinct biological mechanisms in males and females.

Previous studies have demonstrated that, although the incidence of stroke is generally lower in females than in males, females exhibit higher rates of adverse outcomes following stroke (Naveed et al., 2023). Differences in hormone levels, gene expression, and inflammatory response sensitivity between males and females may influence the impact of rs1371097 on stroke prognosis (Bushnell et al., 2014). For instance, estrogen is known to exert neuroprotective effects in females (Villa et al., 2018). However, whether the rs1371097 genetic variant interacts with the estrogen signaling pathway to modulate stroke outcomes remains to be fully elucidated. Exploring this potential mechanism could provide valuable insights into the observed gender disparities in stroke prognosis and should be a focus of future research.

In this study, we combined rs1045642, rs1371097, and relevant clinical factors (including age, gender, history of diabetes, history of cerebral infarction, TOAST classification, admission NIHSS score, fasting blood glucose, red blood cells, CRP, neutrophils, lymphocytes) to construct a relatively reasonable molecular prediction model, which showed a higher predictive efficacy for adverse outcomes of stroke compared to single factors. Considering the complexity of genetic and non-genetic factors in the occurrence and development of clinical outcomes, the impact of individual gene variants on clinical outcomes is relatively small. Moreover, while no single factor can fully predict risk or prevent adverse outcomes, our findings highlight the importance of integrating multiple genetic and clinical factors to improve prognostic accuracy. Therefore, we recommend adopting the strategy used in current research, which is to construct a predictive scoring system to evaluate the long-term treatment with aspirin in acute stroke patients. This comprehensive assessment approach can consider the combined effects of multiple factors, thus more accurately assessing the patient’s risk and guiding the formulation of clinical strategies.

However, this study has several limitations. First, as a single-center retrospective study involving only the Han Chinese population in China, the generalizability of our findings to other ethnic groups remains uncertain and requires further validation. Second, this study focused on acute ischemic stroke patients taking aspirin, but there were no strict restrictions on the concurrent use of other antiplatelet drugs. The interactions between genetic variants and other medications have not been thoroughly investigated and warrant stratified analysis in future studies. Third, although we collected comprehensive data on 90-day modified Rankin Scale (mRS), NIHSS scores, cardiovascular and cerebrovascular composite events, and mortality during follow-up, platelet reactivity testing was unavailable for a significant number of patients. Thus, caution is needed when interpreting the mechanisms by which genetic variants influence stroke prognosis. Fourth, a notable limitation involves the single nucleotide polymorphism (SNP) rs1045642, which did not achieve Hardy-Weinberg equilibrium (HWE) in this cohort. While this deviation may reflect the potential role of this locus in disease pathogenesis, HWE is a fundamental assumption in population genetics, and results related to this SNP should be interpreted with caution. Larger studies with diverse populations are needed to clarify the role of rs1045642 in stroke outcomes. Finally, to minimize the risk of false positives due to multiple comparisons, we applied Bonferroni correction. Unfortunately, none of the genetic variants reached statistical significance after correction, likely due to the limited sample size, small effect sizes, or genetic heterogeneity. Future studies with larger cohorts and integrated genetic and clinical data are essential to validate these associations and provide a more comprehensive understanding.

Despite these limitations, our study proposes a feasible polygenic pharmacogenetic risk score and highlights the potential association between specific genetic variants and adverse prognosis in stroke patients treated with aspirin. Further investigation of these genetic variants may contribute to the development of personalized treatment strategies, improving drug efficacy and reducing adverse outcomes.

## Conclusion

This study suggests that the rs1045642GG genotype is associated with a lower risk of unfavorable outcomes, while the rs1371097T allele is associated with a higher risk in acute ischemic stroke patients receiving aspirin treatment. These SNPs, along with relevant clinical factors, can be incorporated into a predictive scoring system to assess long-term prognosis in this patient population. While further validation is needed, our findings highlight the potential of pharmacogenetic approaches to guide personalized antiplatelet therapy, potentially improving outcomes for stroke patients.

## Data Availability

The original contributions presented in the study are publicly available. This data can be found here: https://bigd.big.ac.cn/gsa-human/browse/HRA010809, accession number HRA010809.
